# Comprehensive analysis of cellular senescence-related genes in the prognosis, tumor microenvironment, and immunotherapy/chemotherapy of clear cell renal cell carcinoma

**DOI:** 10.3389/fimmu.2022.934243

**Published:** 2022-09-16

**Authors:** Caibao Lu, Yiqin Wang, Ling Nie, Liping Chen, Moqi Li, Huimin Qing, Sisi Li, Shuang Wu, Zhe Wang

**Affiliations:** ^1^ Department of Nephrology, The Key Laboratory for the Prevention and Treatment of Chronic Kidney Disease of Chongqing, Chongqing Clinical Research Center of Kidney and Urology Diseases, Xinqiao Hospital, Third Military Medical University (Army Medical University), Chongqing, China; ^2^ Department of Nephrology, Southwest Hospital, Third Military Medical University (Army Medical University), Chongqing, China; ^3^ Department of Oncology and Southwest Cancer Center, Southwest Hospital, Third Military Medical University (Army Medical University), Chongqing, China; ^4^ Key Laboratory of Tumor Immunopathology of Ministry of Education of China, Institute of Pathology and Southwest Cancer Center, Southwest Hospital, Third Military Medical University (Army Medical University), Chongqing, China

**Keywords:** cellular senescence, clear cell renal cell carcinoma, tumor microenvironment, prognostic model, immunotherapy, chemotherapy

## Abstract

**Background:**

The transcriptome public database and advances in biological discoveries contributed to significant progresses in identifying the drivers of cancer progression. Cellular senescence (CS) is considered as a leading factor resulting in cancer development. The purpose of this study was to explore the significance of CS-related genes in the molecular classification and survival outcome of clear cell renal cell carcinoma (ccRCC).

**Methods:**

CS-related genes were obtained from the CellAge database, and patients from TCGA-KIRC dataset and ICGC dataset were clustered by ConsesusClusterPlus. The characteristics of overall survival (OS), genomic variation, and tumor microenvironment (TME) of each cluster were analyzed. Least Absolute Shrinkage and Selection Operator (LASSO) Cox regression analysis was conducted to develop a CS-related risk model to score ccRCC patients and assess the risk scores in predicting patients’ response to immunotherapy and chemotherapy. A nomogram based on the risk model was established to improve the risk stratification of patients.

**Results:**

CcRCC was divided into three molecular subtypes based on CS-related genes. The three molecular phenotypes showed different OS and clinical manifestations, mutation patterns, and TME states. Five genes were obtained from nine differentially expressed CS-related genes in the three molecular subtypes to develop a risk model. Patients with ccRCC were divided into high- and low-risk subgroups. The former showed an unfavorable OS, with a significantly higher genomic variation rate, TME score, and numerous immune checkpoint expressions when compared to the low-risk subgroup. Risk score reflected the response of patients to axitinib, bortezomib, sorafenib, sunitinib, and temsirolimus.

**Conclusions:**

In general, CS-related genes divided ccRCC into three molecular subtypes with distinct OS, mutation patterns, and TME states. The risk model based on the five CS-related genes can predict the prognosis and therapeutic outcome of ccRCC patients, providing a theoretical basis for further study on the molecular mechanism of CS-related ccRCC.

## Introduction

Renal cell carcinoma (RCC) is a fatal cancer of the genitourinary system caused by renal epithelial cells, mainly including three subtypes, namely, clear cell RCC (ccRCC), papillary RCC (pRCC), and chromophobe RCC (chRCC) (1, 2). CcRCC is the most common histological subtype that contributes to about 70% of all RCC cases (3). A clear morphological marker of ccRCC is the accumulation of large amounts of fat and glycogen in the cytoplasm of tumor cells (4). Survival of cancer patients is highly dependent on the stage when diagnosed. Specifically, the 5-year relative survival rate for stage I, stage II/III regional disease (local lymph node involvement), and stage IV with metastatic disease is 93%, 72.5%, and 12%, respectively (5). In a population-based study, after patients received nephrectomy treatment, the 5-year survival for stage I, II, III, and IV RCC was improved to 97.4%, 89.9%, 77.9%, and 26.7%, respectively (6). This indicated that surgical resection with a therapeutic purpose has the potential to treat patients with localized diseases, but 20%–30% of cases relapse within 5 years, usually with metastatic diseases (7). In recent years, tyrosine kinase inhibitor (TKI) cabozantinib and the immunotherapy combination of nivolumab have been considered as the first-line treatment for advanced ccRCC (8). Risk stratification is an important part of clinical trial design in ccRCC, and risk status often guides first-line treatment choice (8). Different models have been developed for patients with local and metastatic diseases. Two of the most commonly used models are the Memorial Sloan Kettering Cancer Center (MSKCC) model and International Metastatic Renal Cell Carcinoma Database Consortium (IMDC) model (9). The MSKCC model divides patients with metastatic RCC into three risk groups based on five characteristics associated with shorter survival, namely, low Karnofsky performance status (<80%), high serum lactate dehydrogenase (>1.5 times upper limit of normal), low hemoglobin (<lower limit of normal), high “corrected” serum calcium (>10 mg/dl), and absence of prior nephrectomy (10). The IMDC model segregated patients with metastatic RCC receiving first-line VEGF-targeted therapy into three risk categories according to the risk factors, including anemia, thrombocytosis, neutrophilia, hypercalcemia, Karnofsky performance status <80%, and <1 year from diagnosis to treatment (11). Although MSKCC and IMDC models are as effective as prognostic markers in clinical practice, their prediction of treatment sensitivity is less accurate. Therefore, the identification of alternative markers to classify tumors based on their major genetic characteristics and molecular pathways has long been the focus of RCC study (12).

Cellular senescence (CS) is an irreversible state of growth arrest, which can be triggered by a variety of mechanisms, including telomere shortening, epigenetic disinhibition of INK4a/ARF loci, and DNA damage. Together, these mechanisms limit excessive or abnormal cell proliferation so that CS can suppress cancer development (13). At present, the identification and characterization of key features of senescence, the induction of senescence in cancer cells, or the elimination of senescent cells through pharmacological interventions in aging tissues are gaining increasing attention from researchers (14). Although CS has an intrinsic tumor suppressor effect, senescent cells are also considered as active contributors to tumorigenesis through externally promoting many markers of cancer, including evading the immune system (15). Therefore, it is necessary to better understand the effects of CS on tumors.

This study classified ccRCC by analyzing known key regulatory factors of CS and established a risk model and a nomogram based on genes related to CS. Moreover, the performance of the risk model for predicting the prognosis of ccRCC, its relationship with tumor variation and tumor microenvironment (TME), and patients’ response to immunotherapy and chemotherapeutic drugs were analyzed.

## Materials and methods

### Obtaining and preprocessing of ccRCC clinical data and cellular senescence-related gens

The KIRC dataset was retrieved from The Cancer Genome Atlas (TCGA, https://portal.gdc.cancer.gov/), and samples with incomplete clinical data records were eliminated. The transcriptional spectrum and clinical data of the remaining 526 samples were included in the training set. The verification cohort containing 91 ccRCC samples was downloaded from the International Cancer Genome Consortium (ICGC, https://dcc.icgc.org/projects/LIRI-JP) database. CS-related genes were obtained from the CellAge (https://genomics.senescence.info/cells/) database, which contained 279 entries, and the majority of genes were associated with replicative senescence (232 genes), stress-induced senescence (34 genes), and oncogene-induced senescence (28 genes) (16). After obtaining the data, we followed the steps in the flowchart (**Supplementary Figure 1**).

### Consensus clustering analysis

Employing the ConsesusClusterPlus package (http://www.bioconductor.org/) in R, consensus clustering analysis was carried out using CS-related genes to classify ccRCC patients. During the analysis, the “partition around medoids” algorithm was used to measure the distance with “Canberra”. The resampling rate was set to 80%, and the bootstraps was set to 500. The number of clusters (2–10) was determined by the consistency matrix and consensus clustering c (CDF).

### Somatic mutation analysis

The Mutation Annotation Format (MAF) downloaded from TCGA was resolved using the “maftools” package. Genomic changes were evaluated by analyzing homologous recombination defects (HRDs), aneuploidy score, fraction altered, and number of segments, and tumor mutation burden (TMB) of different ccRCC samples.

### Proportion of immune cells infiltrated in the TME and overall TME score

To evaluate the distribution of immune cell infiltration in the TME, the proportion of 22 immune cells in each sample was calculated by CIBERSORT. The TME could also be evaluated by calculating the stromal score and immune score and ESTIMATE score of each sample using ESTIMATE. The results were converted into a box chart, with a higher stromal score/immune score indicating more matrix/immune components.

### Construction and verification of a cellular senescence-related risk model

The expression differences of CS-related genes in each of the two molecular subtypes were analyzed by Limma. FDR <0.05 and | log2FC | >1 were defined as the cutoff values. Least Absolute and Selection Operator (LASSO) regression analysis and stepwise multivariable Cox regression analysis were conducted on DEGs to build CS-related risk models according to the following formula: risk score = sum of coefficients × expression level of prognosis CS-related genes. Based on the formula, the risk score of samples in TCGA-KIRC and ICGC was obtained and normalized. Patients were divided into high-risk subgroup and low-risk subgroup. The Kaplan–Meier survival curve and receiver operating characteristic (ROC) curve were generated using R packet “survminer” and “timeROC”, respectively, to assess the prognostic effect of the risk model.

### Quantitative real-time PCR

Five cell lines were purchased from ATCC, namely, HK-2, 786-O, A489, CAKI-1, and ACHN. Among them, HK-2 is a human renal proximal convoluted tubule cell, and the other four cell lines belong to ccRCC cells. To purify RNA from cell lines, we used TRIzol Reagent (Solarbio, Beijing, China) according to the manufacturer’s instructions. We synthesized cDNA from total RNA using PrimeScript^®^ RT Reagent Kit (Takara, Kusatsu, Japan). The SYBR^®^ Premix Ex Taq (TaKaRa) was used for quantitative real-time PCR (qRT-PCR) assays. GAPDH was chosen as an internal control. All the primers for mRNAs are as follows:

XAF1-F: 5′-CTTACTGCCTGCGGTTCCTG-3′
XAF1-R: 5′-CGTACACCCAACCTGCTGGT-3′
IRF7-F: 5′-TGGTCCTGGTGAAGCTGGAA-3′
IRF7-R: 5′-GATGTCGTCATAGAGGCTGTTGG-3′
NTN4-F: 5′-GTACTTTGCGACTAACTGCTCC-3′
NTN4-R: 5′-TCCAGTGCATGGAAAAGGACT-3′
ETS1-F: 5′-CTGCCCGGGCGGATCCATGAGCTACTTTGTGGATTCTGC-3′
ETS1-R: 5′-CGGTATCGATAAGCTTTCACTCGTCGGCATCTGG-3′
KL-F: 5′-CCAAAGTCTGGCATCTCTACAAC-3′
KL-R: 5′-AGCCTAGCACAAAGTCAAGAGAC-3′
GAPDH-F: 5′-TGCACCACCAACTGCTTAGC-3′
GAPDH-R: 5′-GGCATGGACTGTGGTCATGAG-3′

### Western blot

The cells were washed with washing buffer and lysed in RIPA buffer (R0010, Solarbio, China) containing protease inhibitors (Roche). The protein concentration was detected using bicinchoninic acid (BCA) protein assay kit (Pierce, Rockford, IL). The soluble components mixed with 5× loading buffer were boiled for 5 min. Protein was separated by SDS-PAGE and transferred to a PVDF membrane (Merck Millipore, Billerica, MA).

After being blocked with 5% non-fat milk, the membranes were incubated overnight at 4°C with appropriate dilutions of primary antibodies against XAF1 (13805, Cell Signaling), IRF7 (sc-74471, Santa Cruz), NTN4 (sc-365280, Santa Cruz), ETS1 (14069, Cell Signaling), and KL (ab203576, Abcam). Then, the PVDF membranes were washed with TBST; after that, they were incubated with a corresponding secondary antibody for 2 h. The proteins were identified by Pierce SuperSignal West Pico Chemiluminescent Substrate (Thermo Fisher, Waltham, MA), as described by the manufacturer. A GAPDH antibody was used as an internal reference.

### Prediction of immunotherapy response in risk groups

The potential relationship between risk score and efficacy of immunotherapy was evaluated through analyzing the expression of immune checkpoint molecules, an effective marker of immunotherapy, in different risk scores. Tumor Immune Dysfunction and Exclusion (TIDE, http://tide.dfci.harvard.edu/) is a computational method used to simulate two main mechanisms of tumor immune evasion. The response to immunotherapy was evaluated by calculating the TIDE score of each patient in the risk group.

### Prediction of chemotherapy response in risk groups

The relationship between risk score and chemotherapy was assessed by predicting the correlation between tumor drug resistance-related cell tumor-associated macrophage (TAM), myeloid-derived suppressor cell (MDSC), cancer-associated fibroblast (CAF), and risk score. According to Genomics of Drug Sensitivity in Cancer (GDSC https://www.cancerrxgene.org/), the half-maximal inhibitory concentration (IC50) served as an index to compare the response of risk groups to chemotherapeutic drugs.

### Development of a nomogram

Univariate Cox and multivariate Cox regression analyses were performed on the clinicopathological features obtained from TCGA-KIRC and the risk score so as to screen independent factors significantly related to the prognosis of ccRCC. The “Rms” package was used to develop a nomogram with comprehensive independent prognostic factors. The consistency between nomogram-predicted ccRCC survival and the actual survival results was analyzed by generating a calibration plot. Decision curve analysis was performed to compare the net benefits of different clinical features, nomogram, and risk score.

### Statistical analysis

All Kaplan–Meier survival curves were visualized by the “survminer” package. The “forestplot” package of R was used to display the results of univariate and multivariate Cox regression analyses. The correlation between variables was evaluated by Pearson correlation test. All the bioinformatics data processing was conducted in R 4.0.1 software. If not specified, P < 0.05 indicated a statistical significance.

## Results

### Three molecular subtypes of ccRCC were identified based on CS-related genes

**Figure 1:**
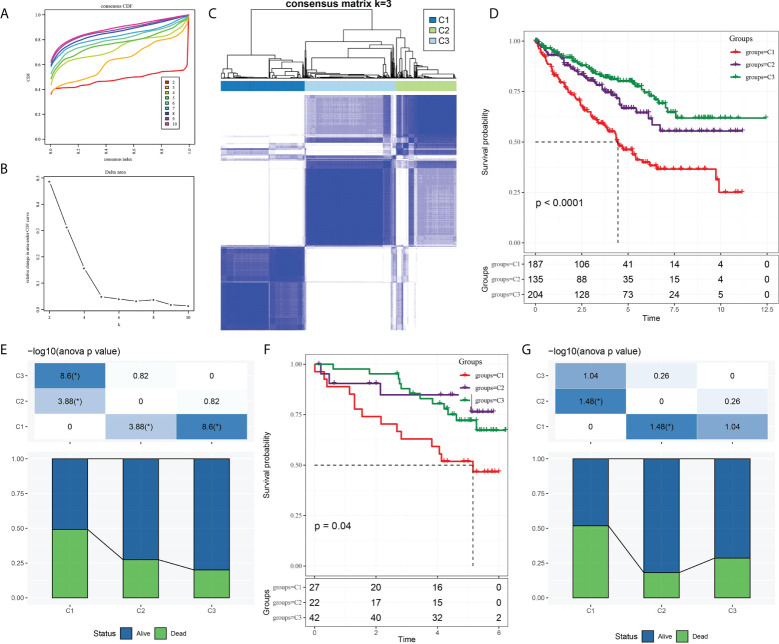
Three molecular subtypes of ccRCC were identified based on CS-related genes. **(A)** Consensus clustering cumulative distribution function (CDF) of k = 2–10. **(B)** The trend under the CDF curve when k = 2 to 9. **(C)** Heatmap depicted sample clustering at consensus k = 3. **(D)** The Kaplan–Meier curve of survival analysis for three molecular subgroups in TCGA-KIRC. **(E)** The proportion of patients who survived and died in each subgroup of TCGA-KIRC. **(F)** The Kaplan–Meier OS curves of three molecular subtypes in ICGC. **(G)** Survival and mortality of each molecular subtype in ICGC.

The expression of CS-related genes was extracted from the expression profile of TCGA-KIRC, and 153 genes associated with ccRCC survival were obtained using univariate Cox regression analysis with coxph function. Five hundred twenty-six samples in TCGA-KIRC were clustered according to the expression of 153 genes. CcRCC was divided into three molecular subtypes, namely, cluster 1 (C1), cluster 2 (C2), and cluster (C3) (**Figures 1A** **–C**). We noticed significant differences in survival rates among the three molecular subtypes of TCGA-KIRC. The highest survival rate was C3 (more than 75%), the middle survival rate was C2 (between 65 and 75%), and the lowest survival rate was C1 (close to 50%) (**Figures 1D, E** **)**. There were also significant differences in survival among the three subtypes of ICGC. It should be pointed out that the 3-year survival rate of the C3 subtype was significantly higher than that of C2 and C1; however, from years 3 to 6, the survival rate of C2 was higher than that of C1 and C3 (**Figure 1F**). Overall, the survival rate of C1 was lower than 50%. The proportion of surviving patients in C2 was higher than 80%. The proportion of surviving patients in C3 was lower than 75% **(** **Figure 1G** **)**.

### Clinical characteristics of the three subtypes

**Figure 2:**
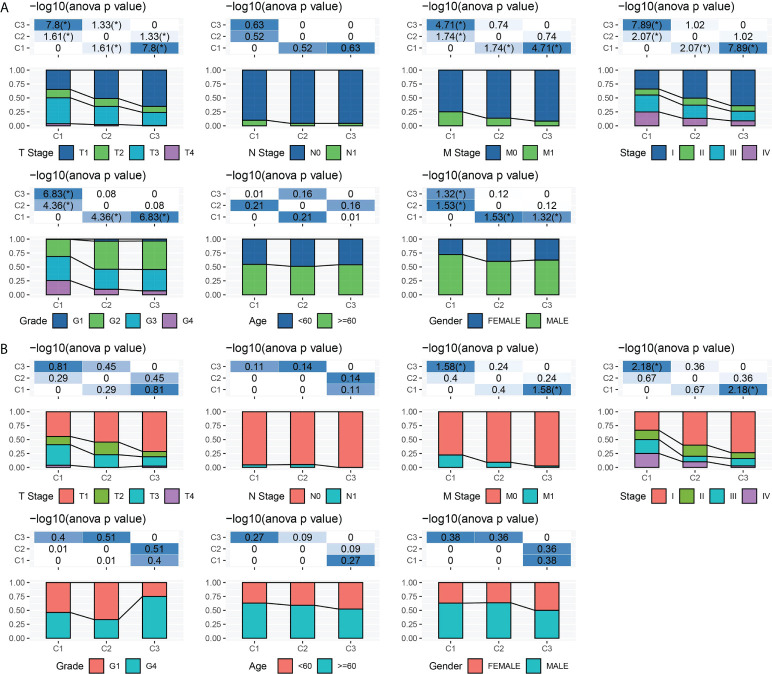
Clinical characteristics of three subtypes. **(A)** T stage, N stage, M stage, pathological stage, grade, age, and gender characteristics of three subtypes in TCGA-KIRC. **(B)** T stage, N stage, M stage, pathological stage, grade, age, and gender characteristics of three subtypes in ICGC. *P < 0.05.

To explore the relationship between molecular subtypes and pathological features of patients with ccRCC, different clinical features of each molecular subtype in TCGA-KIRC and ICGC data sets were analyzed. In the former data set, there were significant differences in T stage, M stage, pathological stage, grade, and gender among the three subtypes. Moreover, C1 patients had a more advanced T stage, M stage, and pathological stage; a higher grade; and a large proportion of male patients (**Figure 2A**). Only significant correlations between molecular subtypes and T stage, M stage, and pathological stage were detected in ICGC. The trend of T stage, M stage, and pathological stage of the three subtypes was the same as that observed in TCGA-KIRC dataset (**Figure 2B**).

### Mutation and TME characteristics of the three subtypes

**Figure 3:**
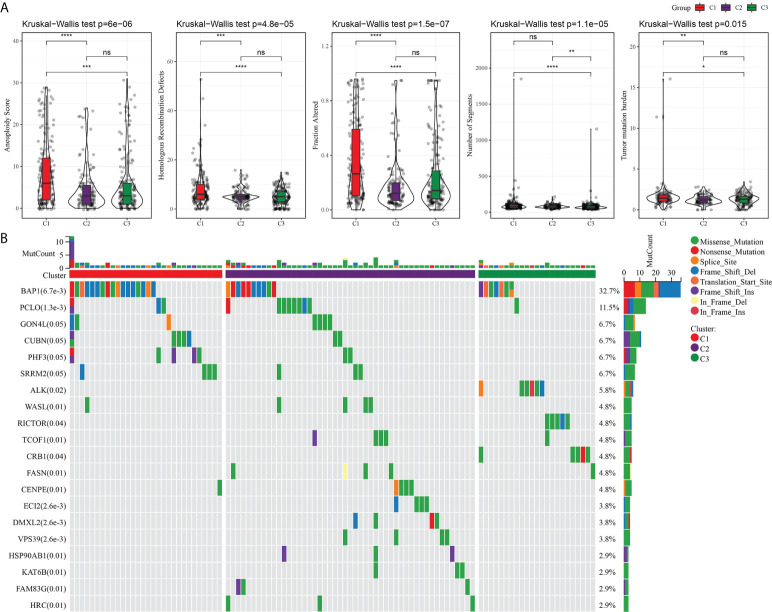
Mutation analysis of three subtypes. **(A)** The difference among three subtypes in aneuploidy score, HRDs, fraction altered, number of segments, and tumor mutation burden. **(B)** Mutation frequency and mutation pattern of the 20 most frequently mutated genes in the three subtypes. *P < 0.05, **P < 0.01, ***P < 0.001, ****P < 0.0001. ns, not significant.

An analysis and comparison of aneuploidy score, HRDs, fraction altered, number of segments, and TMB of the three molecular subtypes showed that there were significant differences in these genomic variation indexes among the three subtypes. C1 had the most prominent aneuploidy score, HRDs, fraction altered, number of segments, and TMB (**Figure 3A**). The waterfall chart revealed that the three subtypes had different mutation patterns. The mutation frequency of BAP1 was the highest. The mutation mode of C1 was mainly frame shift deletion, and the main mutation mode of most genes in C2 and C3 was missense mutation (**Figure 3B**).

**Figure 4:**
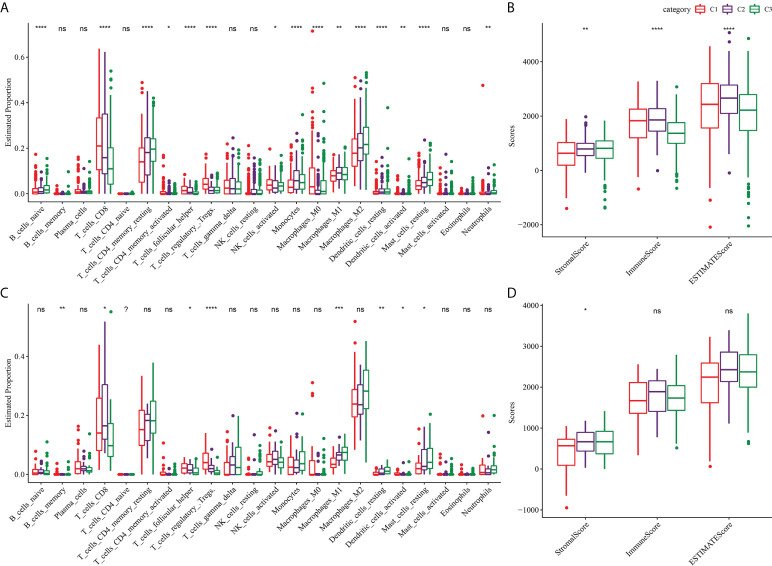
TME characterization of three clusters. **(A)** The proportion of immune cells in the three subtypes of TCGA-KIRC dataset. **(B)** Stromal score and immune score and ESTIMATE score among three subtypes in TCGA-KIRC dataset. **(C)** The proportion of immune cells among the three clusters of the ICGC dataset. **(D)** Differences of stromal score, immune score, and ESTIMATE score among three clusters of ICGC dataset. *P < 0.05, **P < 0.01, ***P < 0.001, ****P < 0.0001. ns, not significant.

Because of the close relationship between TMB and TME, we further analyzed the proportion of immune cells in the three subtypes of TME. For the three subtypes of TCGA-KIRC, the proportions of naive B cells, CD8 T cells, resting memory CD4 T cells, activated memory CD4 T cells, helper follicular T cells, regulatory T cells, activated NK cells, monocytes, M0 macrophages, M1 macrophages, M2 macrophages, resting dendritic cells, activated dendritic cells and resting mast cells, and neutrophils in the TME were significantly different (**Figure 4A**). The stomal score of C1 was noticeably lower compared to C2 and C3, and the immune score and ESTIMATE of C3 were significantly lower than those of C1 and C2. Among the three subtypes of TCGA-KIRC, C1 had the lowest matrix content and C3 had the lowest level of immune cell infiltration and tumor purity (**Figure 4B**). The three subtypes in the ICGC dataset showed significant differences in the proportion of memory B cells, CD8 T cells, helper follicular T cells, regulatory T cells, M1 macrophages, resting dendritic cells, activated dendritic cells, and resting mast cells (**Figure 4C**). Moreover, significant differences in stromal score were found among the three subtypes, and the trend was the same as that of TCGA-KIRC (**Figure 4D**).

### Construction and verification of the CS-related risk model

**Figure 5:**
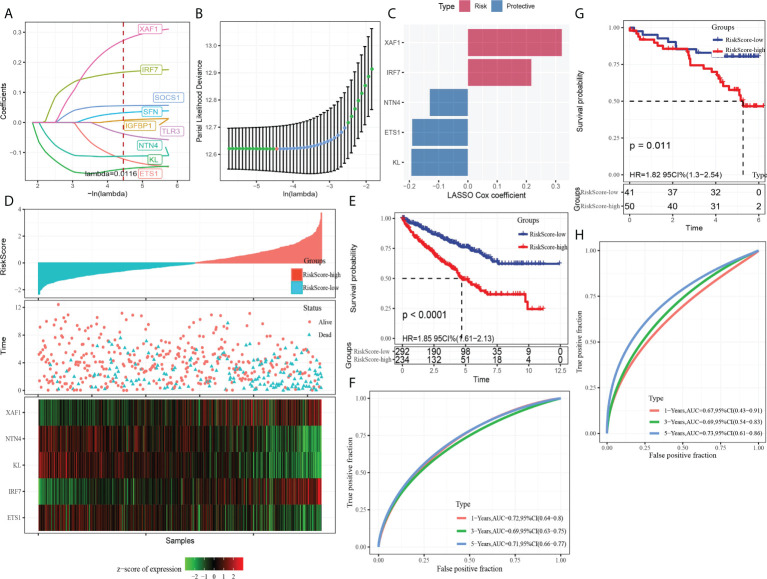
Establishment and verification of the CS-related risk model. **(A)** The LASSO coefficient curves of nine CS-related genes. **(B)** Ten-fold cross-validation of adjusting parameter selection in LASSO regression. **(C)** LASSO Cox coefficient of the five genes. **(D)** The arrangement of risk score, the depiction of survival status, and the expression trend of five genes in the samples of TCGA-KIRC dataset. **(E)** Kaplan–Meier curve of survival of the high-risk group and low-risk group in TCGA-KIRC. **(F)** The ROC curve showed the AUC of risk score predicting the survival of patients in TCGA-KIRC. **(G)** The Kaplan–Meier curve depicts the survival trend of high-risk and low-risk patients in ICGC datasets. **(H)** The ROC curve for patients OS in ICGC was predicted by the risk model.

**Figure 6:**
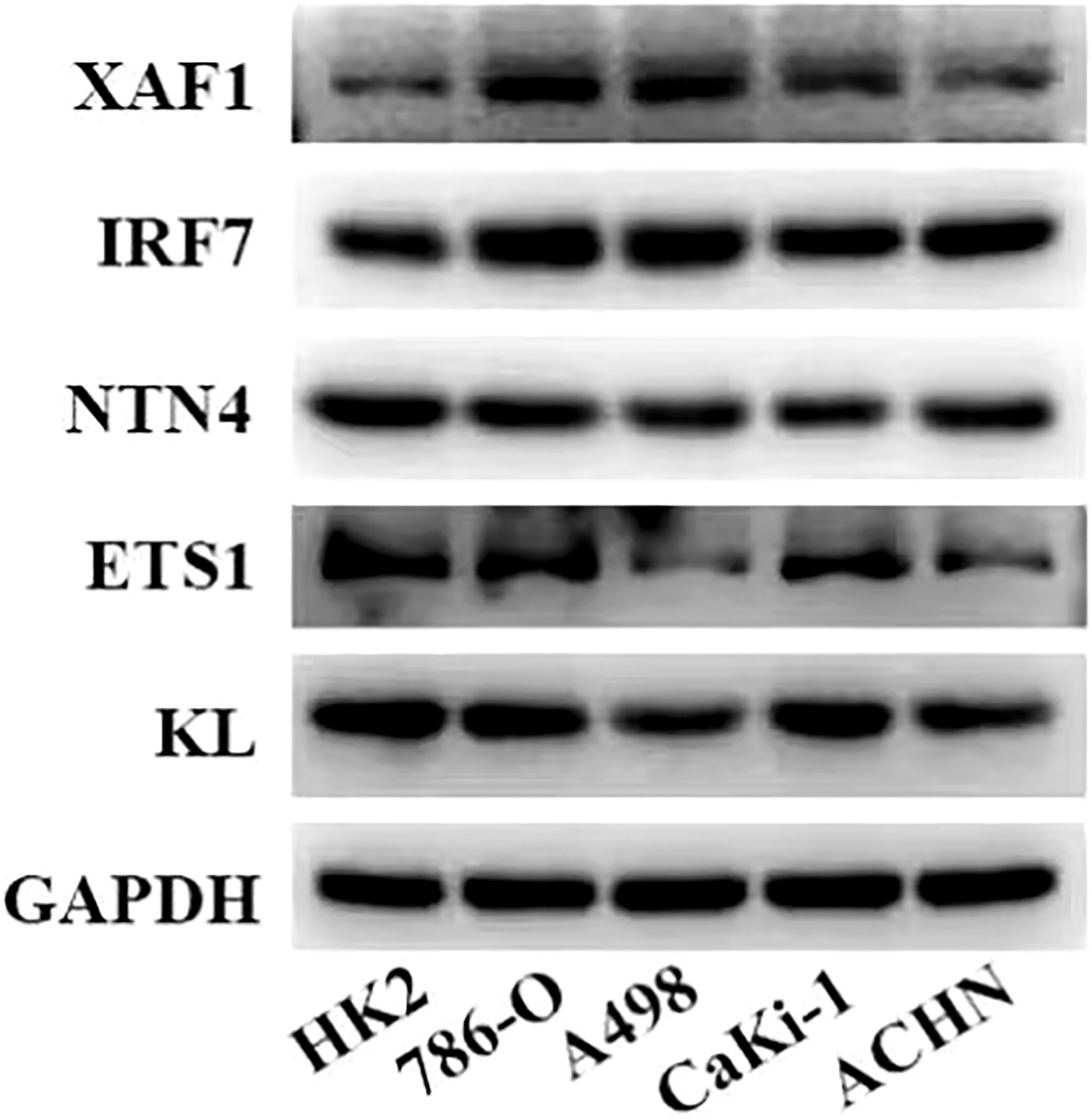
Expression levels of five genes in the risk model in normal kidney cells and ccRCC cells measured by Western blot.

A total of nine DEGs were identified by the difference analysis on CS-related genes between the two molecular subgroups (ETS1, TLR3, KL, NTN4, SFN, IGFBP1, IRF7, SOCS1, XAF1) (**Supplementary Figure 2**). The LASSO regression analysis on the DEGs demonstrated that the coefficients of nine DEGs were all greater than 0 according to the optimal value of λ (**Figures 5A, B** **)**. To screen out the genes with the greatest influence on ccRCC survival from nine DEGs, stepwise multivariate Cox regression was performed, which is a method of multiple regression analysis. Regression analysis is used to study the interdependent relationship between multiple variables, while stepwise regression analysis is often used to establish the optimal or appropriate regression model, so as to further study the dependence relationship between variables. We constructed a multivariate COX regression model for five genes, which were composed of two risk factors (XAF1 and IRF7) and three protective factors (NTN4, ETS1, and KL) (**Figure 5C**). The risk model calculated the risk score of each sample in TCGA-KIRC dataset and depicted the survival status. The death rate of patients in the high-score group increased significantly. For the five genes, the expression of risk factors XAF1 and IRF7 was upregulated with the increase in risk score, while that of protective factors was opposite to that of risk factors (**Figure 5D**). The expression of each gene in the risk model independently predicted the prognosis of ccRCC. The high expression of each protective factor represented a higher OS in the ccRCC sample, while the high expression of each risk factor indicated a poor prognosis in the ccRCC sample (**Supplementary Figure 3**). Besides, qRT-PCR and Western blot were also performed to verify the mRNA levels and expression levels of the five genes. Compared with normal HK-2 cells, the transcription and expression levels of XAF1 and IRF7 in ccRCC cells were significantly increased, while NTN4, ETS1, and KL were significantly decreased (**Figure 6** and **Supplementary Figure 4**). For patients in TCGA-KIRC, the OS of the low-risk score subgroup was significantly higher than that of the high-risk score subgroup (**Figure 5E**). The ROC curve showed that the AUC value of risk score for predicting 1-, 2-, and 3-year survival rates was 0.72, 0.69, and 0.71, respectively (**Figure 5F**). The risk model was used to predict the survival of patients in ICGC. The results showed that the long-term survival rate of patients with a low-risk score was significantly higher than that of patients with a high-risk score (**Figure 5G**). The prediction efficiency of the model in the ICGC cohort was also high, with AUC values of 0.67,0.69, and 0.73 for 1, 3, and 5 years, respectively (**Figure 5H**).

### The risk score characterized by distinct mutation profiles

**Figure 7:**
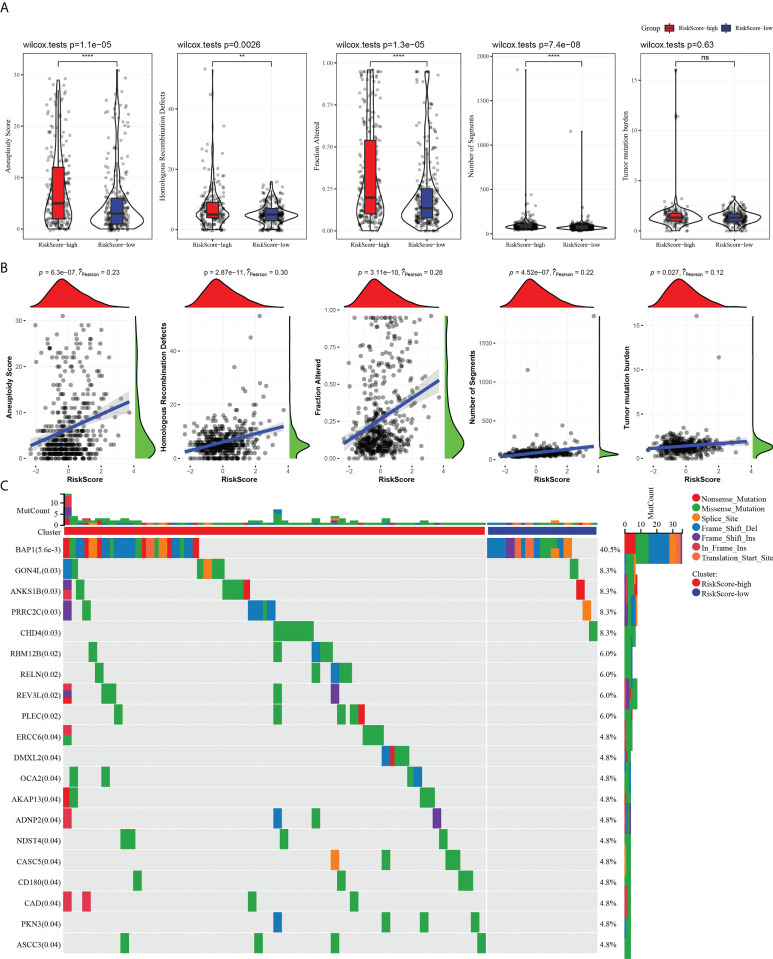
The risk score characterized by distinct mutation profiles. **(A)** Aneuploidy score, HRDs, fraction altered, number of segments, and TMB between high-risk and low-risk subgroups. **(B)** Correlation analysis between risk score and aneuploidy score, HRDs, fraction altered, number of segments, tumor mutation burden. **(C)** Distribution of gene mutations in the high-risk and low-risk subgroups. **P < 0.01, ****P < 0.0001. ns, not significant.

HRDs, fraction altered, number of segments, and TMB in high- and low-risk groups were analyzed o describe the genomic abnormalities in the risk model established based on CS-related genes, aneuploidy score. Aneuploidy score, HRDs, fraction altered, and number of segments in the high-risk group were significantly higher than those in the low-risk group (**Figure 7A**). The correlation between risk score and these variation types was high, and the coefficients were greater than 0 (**Figure 7B**). The somatic mutations in the high-risk subgroup were more widely distributed, and the mutation rates were relatively higher compared to the low-risk subgroup (**Figure 7C**). The results showed that the risk model could effectively reflect the genomic mutation characteristics of ccRCC.

### The risk score characterized by distinct immune profiles

**Figure 8:**
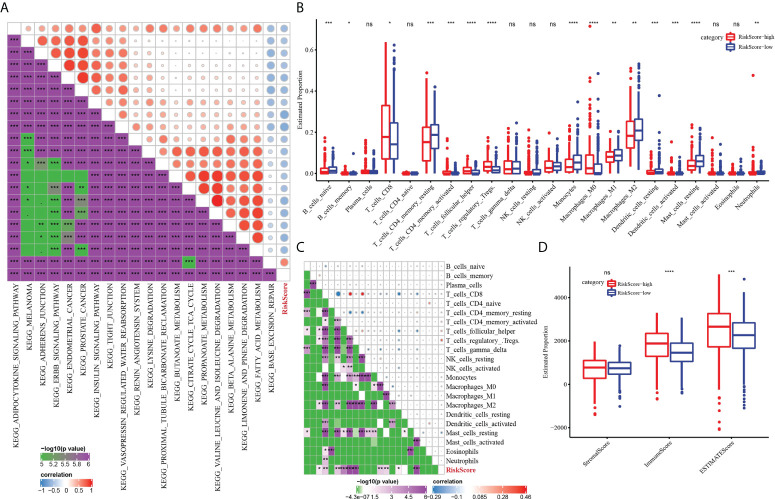
The immune characteristics of ccRCC were characterized by the risk model. **(A)** The relationship between risk score and KEGG signal pathway. **(B)** Analysis for the difference in the proportion of 22 kinds of immune cells between the high-risk subgroup and low-risk subgroup. **(C)** Pearson correlation between risk score and 22 kinds of immune cells. **(D)** The difference of three TME-related scores between the high-risk subgroup and low-risk subgroup. *P < 0.05, **P < 0.01, ***P < 0.001, ****P < 0.0001. ns, not significant.

We further described the biological pathway of risk score mediated by CS-related genes and analyzed the relationship between risk score and KEGG signal pathway. **Figure 8A** shows that risk score was positively correlated with base excision repair but was negatively correlated with metabolic regulation signals (fatty acid metabolism, propanoate metabolism, butanoate metabolism, citrate cycle TCA cycle, valine leucine isoleucine degradation) and metastasis-related signals (adherens junction and tight junction). The difference analysis of 22 kinds of immune cell proportion between the high-risk group and the low-risk group showed that the proportion of memory B cells, CD8 T cells, activated memory CD4 T cells, helper follicular T cells and regulatory T cells, and M0 macrophages in the high-risk score subgroup was significantly higher than that in the low-risk score group, while the proportion of naive B cells, resting memory CD4 T cells, monocytes, M1 macrophages, M2 macrophages, and resting dendritic cells and resting mast cells was noticeably reduced in the high-risk score group (**Figure 8B**). The results of Pearson correlation analysis demonstrated the relation between risk score and plasma cells, CD8 T cells, resting memory CD4 T cells, activated memory CD4 T cells, helper follicular T cells, regulatory T cells, monocytes, M0 macrophages, M2 macrophages, and resting mast cells (**Figure 8C**). In addition, patients with a high-risk score showed a higher immune score and ESTIMATE score than those with a low-risk score (**Figure 8D**).

### Patients’ response to immunotherapy and chemotherapy predicted by the risk model

**Figure 9:**
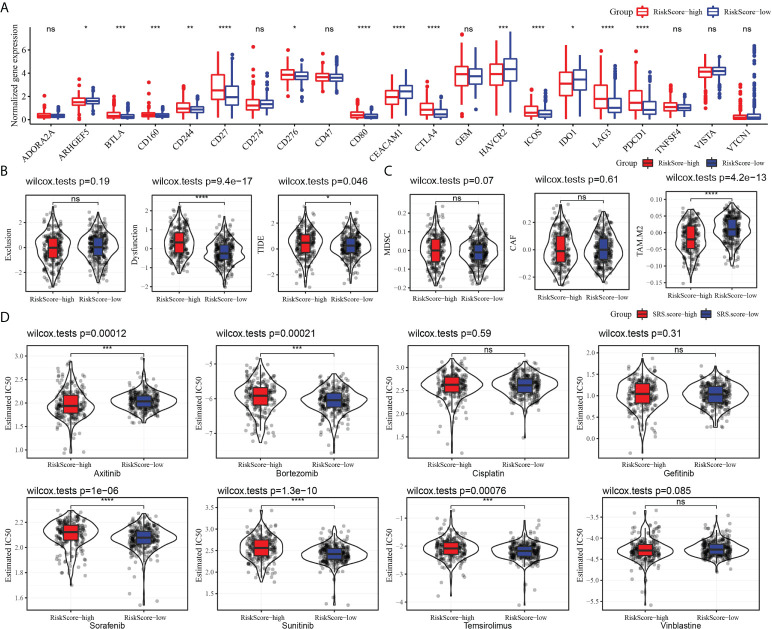
Risk model predicted response to immunotherapy and chemotherapy. **(A)** The level of 21 immune checkpoints in the high-risk subgroup and low-risk subgroup. **(B)** The relationship between risk score and tumor immune exclusion, tumor immune dysfunction, and TIDE. **(C)** Association of risk score with chemotherapy-resistant cells MDSC, CAF, and M2 TAM. **(D)** Sensitivity of eight types of chemotherapeutic drugs in high-risk and low-risk subgroups. *P < 0.05, **P < 0.01, ***P < 0.001, ****P < 0.0001, ns, not significant.

We evaluated the relationship between risk model and immune checkpoint expression. Fourteen out of the 21 immune checkpoints were detected to be differentially expressed between the high-risk subgroup and the low-risk subgroup. It should be noted that the expression of CTLA4 and PDCD1 in the high-risk group was significantly higher than that in the low-risk group. Additionally, T-cell response markers (LAG3 and BLTA) were also highly expressed in high-risk groups (17), indicating that the risk model had the ability to identify potential immune disorders (**Figure 9A**). Then, the relationship between risk score and immunotherapy response was investigated. The high-risk subgroup showed higher tumor immune dysfunction and TIDE, indicating a higher probability of potential immune escape in the TME of the high-risk group (**Figure 9B**). The value of risk score in predicting the response to chemotherapy was also explored. Among the three tumor immune cells associated with chemotherapy resistance, the level of M2 TAM in the high-risk subgroup was significantly lower than that in the low-risk group (**Figure 9C**). Eight clinical chemotherapeutic drugs were selected to analyze their sensitivity in high-risk and low-risk subgroups. Compared with the IC50 value of the two groups, the high-risk subgroup responded more actively to axitinib. The low-risk subgroup responded better to bortezomib, sorafenib, sunitinib, and temsirolimus (**Figure 9D**).

### The nomogram improved the prognostic value of the CS-related risk model

**Figure 10:**
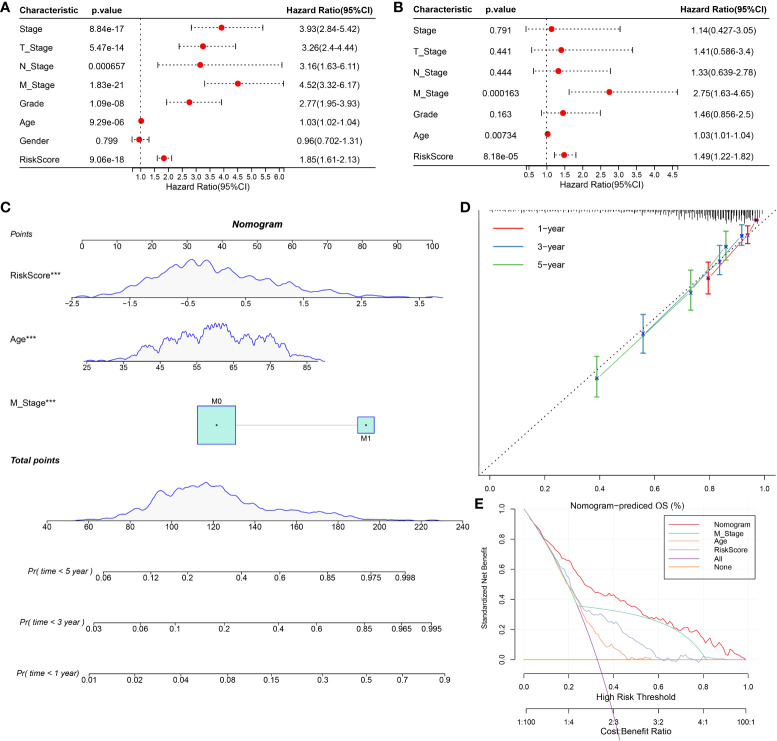
Construction and evaluation of nomogram based on independent prognostic indicators of ccRCC. **(A, B)** Univariate and multivariate Cox regression analyses screened the independent prognostic indicators of ccRCC from the clinical features of ccRCC and risk score. **(C)** Construction of a nomogram in conjunction with variables that independently predict ccRCC prognosis. **(D)** The calibration plot of the nomogram. **(E)** Decision curve analysis for ccRCC prognosis-related index and nomogram.

To screen the independent prognostic factors of ccRCC, univariate and multivariate Cox regression analyses were performed. M stage, age, and risk score had independent prognostic values in ccRCC (**Figures 10A, B** **)**. A nomogram combining these independent prognostic variables of ccRCC was developed (**Figure 10C**). The prediction lines of 1-, 3-, and 5-year survival of the nomogram were close to the 45° dotted line of calibration analysis, indicating that its prediction performance was ideal (**Figure 10D**). The decision curve showed that the nomogram had a higher net income than other prognostic indicators of ccRCC (**Figure 10E**). These results suggested that the nomogram improved the predictive performance of the risk model and was a potentially ideal model for predicting the prognosis of ccRCC.

## Discussion

CcRCC is a group of highly heterogeneous renal tumors developed from different genetic and epigenetic drive mechanisms and molecular pathways. As a result, patients’ responses to treatment vary widely, increasing the additional complexity to the already challenging decision-making process in treatment (12). A detailed pathological classification of ccRCC is highly needed currently. The identification of specific markers with the highest sensitivity will induce more biologically oriented tumor classification, which will facilitate treatment at all stages of tumorigenesis (18). Publicly available comprehensive molecular marker libraries offer high-quality resources for the current research. CellAge is a database that stores CS-related genes. We analyzed the genes included in this database and identified three molecular subtypes related to the CS of ccRCC. The three molecular phenotypes showed different prognoses and clinical manifestations, mutation patterns, and TME status.

The molecular characteristics of the initiation and progression of ccRCC are increasingly defined. The TRACERx kidney study and other studies describing the interaction between tumor genomics and tumor microenvironment remodeling provide important new insights into the molecular drivers of ccRCC ontogeny and progression (3). CS-related genes have also been used to characterize the clinical prognosis of a variety of cancers, such as diffuse gliomas (19), lung adenocarcinoma (20), and gastric cancer (21). In this study, considering the heterogeneity of ccRCC, we established a risk model based on DEGs filtered from CS molecular subtypes. Although the research procedure of ccRCC in this study is similar to the new study published by Zhou et al. (22), the risk model established was different. The risk model constructed by Zhou is composed of nine genes P3H1, PROX1, HJURP, HK3, CDKN1A, AR, VENTX, MAGOHB, and MAP2K6, while the risk model constructed by our study included five genes (XAF1, IRF7, NTN4, ETS1, and KL).

Senescent cells secrete a variety of proteins, such as inflammatory cytokines, chemokines, and growth factors, which are important components of the TME (23). Moreover, senescent cells can reshape surrounding tissue by regulating the properties of neighboring cells, including stromal and immune cells (24). Therefore, CS-related genes may affect the TME. Additionally, pan-cancer analysis showed that ccRCC was one of the tumors with the greatest immune infiltration (25). Therefore, we also analyzed the TME effect of CS-related genes on ccRCC. Risk models established using CS-related genes were associated with the proportion of most immune cells. In the high-risk score subgroup, the proportion of memory B cells, CD8 T cells, activated memory CD4 T cells, helper follicular T cells, and regulatory T cells and M0 macrophages was significantly higher, while that of naive B cells, resting memory CD4 T cells, monocytes, M1 macrophages, M2 macrophages, and resting dendritic cells and resting mast cells was significantly reduced.

Another remarkable feature of ccRCC is great changes in cell metabolism (4). The results of this study showed that the risk model was closely associated with a variety of metabolic biological pathways, such as fatty acid metabolism, propanoate metabolism, butanoate metabolism, citrate cycle TCA cycle, and valine leucine isoleucine degradation. Previous reports mentioned that truncal mutations in ccRCC including mutations in VHL, SET2, PBRM1, and BAP1 may lead to genomic instability and promote defects in the DNA repair pathway (3). Here, the BAP1 mutation frequency was the highest in the high-risk group, and DNA damage-related patterns including aneuploidy score, HRDs, fraction altered, number of segments, and TMB were associated with the high-risk group. Genomic instability is one of the important markers of cancer including ccRCC, which is a feature that promotes cancer progression and resistance to treatment (26). Higher HRD scores were associated with poorer outcomes for several cancers (27). In addition, markers of genomic stability including fraction altered, number of segments, and TMB usually have a consistent trend in cancer (28). Four of the markers here assessed for genomic instability were significantly higher in the high-risk group, providing evidence for poor outcomes in the ccRCC samples in the high-risk group.

The characteristics of the TME could affect disease biology as well as the response to systemic therapy (25). Here, we detected a significant increase in the expression of CTLA4 and PDCD1 in the high-risk group. In addition, T-cell response markers LAG3 and BLTA were also highly expressed in the high-risk subgroup, indicating that the risk model had the ability to identify potential immune disorders. Furthermore, correlation analysis of risk score and tumor immune dysfunction and TIDE confirmed the existence of immune escape in ccRCC. Interestingly, our analysis also showed that risk score was related to the tumor immune cell level associated with drug resistance.

The genes in the risk model should also be more comprehensively studied. XAF1 has been reported as a prognostic biomarker and therapeutic target for lung squamous cell carcinoma (29) and pancreatic cancer (30). AXF1 has previously been found to contribute to endothelial cell senescence (31). Interferon regulatory factor 7 (IRF7) was overexpressed in gastric adenocarcinoma, which was significantly correlated with poor OS and immune infiltration (32). The reactivation of IFN-regulated genes by transcription factors IRF7 is sufficient to induce cellular senescence (33). IRF7 was involved in the immune response of low-grade glioma to influence tumor progression (34). The expression of nerve guidance factor 4 (NTN4), which is a regulatory molecule of epithelial–mesenchymal transformation in breast adenocarcinoma, was reduced in breast cancer samples (35). More importantly, NTN4 protected glioblastoma cells from drug temozolomide-induced senescence, indicating its effect on tumor CS (36). KL deficiency reduces telomerase activity by altering the expression of TERF1 and TERT, leading to stem cell senescence (37). Ets1 belongs to the large family of the ETS domain family of transcription factors and is associated with poor prognosis in most cancers (38). ETS1 has already been reported to be strongly associated with the aging of 27 different tissues in two different species (39). These CS-related genes in the risk model played important roles in a variety of cancers, and their combination in one model was acceptable.

There are several limitations to this study. First, we only considered heterogeneity between ccRCC samples and ignored heterogeneity within tumors. Secondly, the sample size of the study is relatively limited and lacks the support of a large amount of data. In addition, the results still need further experimental verification.

In brief, this study analyzed the molecular classification of ccRCC based on CS-related genes and revealed the clinical characteristics, prognosis, and TME characteristics of different molecular categories. A new molecular model related to CS was developed, which could effectively reflect the genomic mutation and TME characteristics of ccRCC and the effect of immunotherapy/chemotherapy. The current model should be further explored and may provide some novel insights into the study of the regulatory mechanism underlying the CS in ccRCC.

## Data availability statement

The datasets presented in this study can be found in online repositories. All data relevant to the study are included in the article or uploaded as online supplemental information.

## Author contributions

ZW, SW, and CL designed the research. CL, YW, LN, LC, ML, HQ, SL, and SW collected and analyzed the data. CL, YW, and LN drafted the manuscript. ZW and SW reviewed and revised the manuscript. All authors contributed to the article and approved the submitted version.

## Funding

This study was supported by the Appropriate Health Technology Promotion Project of Chongqing (2020jstg030), Science and Technology Innovation Projects for Social Undertakings and Livelihood Security of Chongqing (cstc2017shmsA130106), Clinical Medical Research Talents Training Program of Army Medical University (2018XLC3029), and National Natural Science Foundation of China (nos. 81472698).

## Acknowledgments

We thank all of the researchers who have uploaded and shared their databases to make this work possible.

## Conflict of interest

The authors declare that the research was conducted in the absence of any commercial or financial relationships that could be construed as a potential conflict of interest.

## Publisher’s note

All claims expressed in this article are solely those of the authors and do not necessarily represent those of their affiliated organizations, or those of the publisher, the editors and the reviewers. Any product that may be evaluated in this article, or claim that may be made by its manufacturer, is not guaranteed or endorsed by the publisher.

## Supplementary material

Supplementary Figure 1 The flowchart of this study.

Supplementary Figure 2 The Venn diagram shows the the number of DEGs between each of the two molecular subtypes.

Supplementary Figure 3 Survival curves based on the expression of each CS-related gene in the risk model.

Supplementary Figure 4 mRNA levels of five genes in the risk model in normal kidney cells and ccRCC cells measured by qRT-PCR. Data are shown as mean ± SD, *p<0.05, **p<0.01, ***p<0.001.
