# Supplementary figures and images for: Comprehensive analysis of cellular senescence-related genes in the prognosis, tumor microenvironment, and immunotherapy/chemotherapy of clear cell renal cell carcinoma
Source: Front Immunol. 2022 Sep 16;13:934243. doi: 10.3389/fimmu.2022.934243 (PMC9523431; doi:10.3389/fimmu.2022.934243)

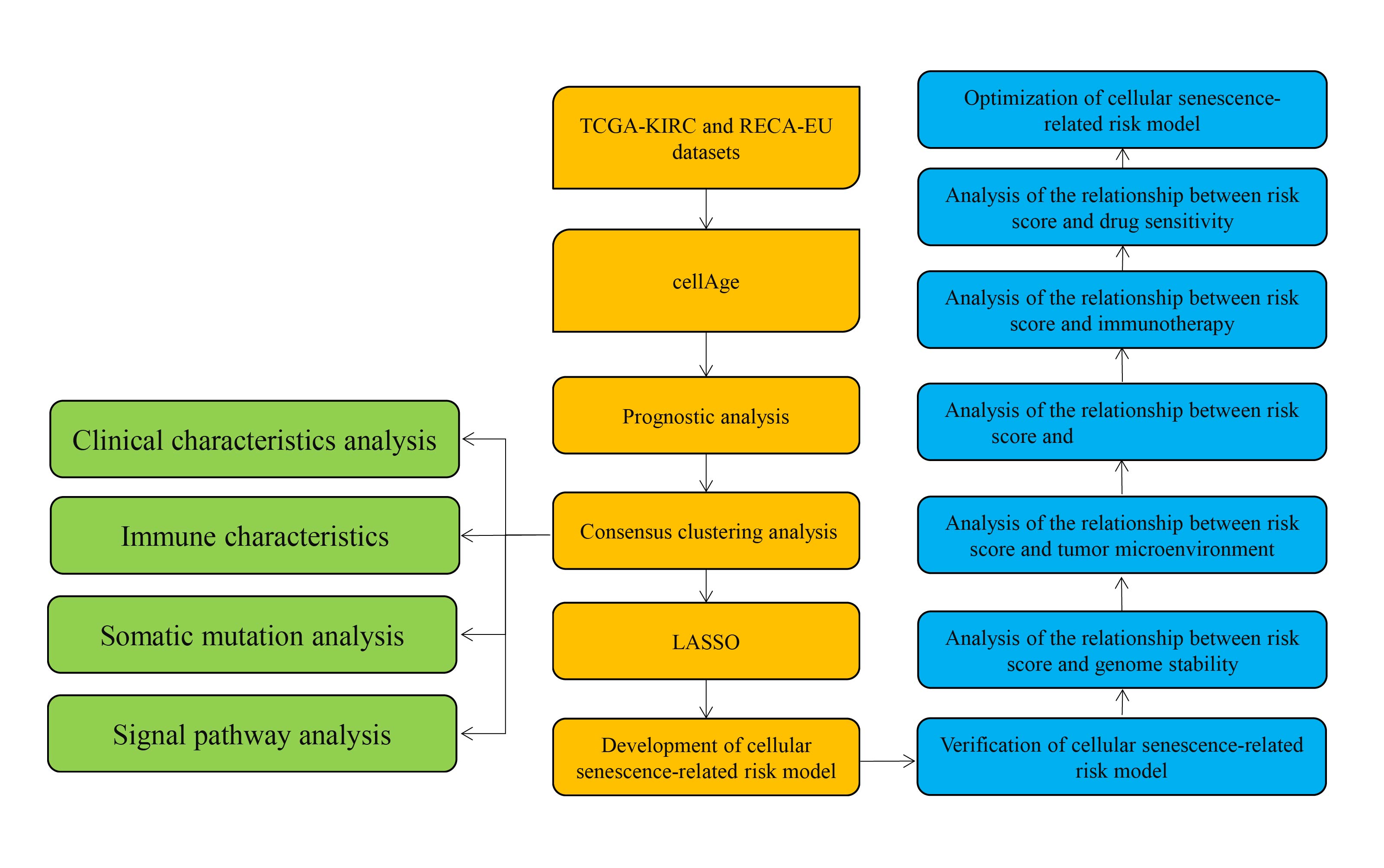

Supplement: Supplementary Figure 1 — The flowchart of this study. [file Image_1.jpeg]

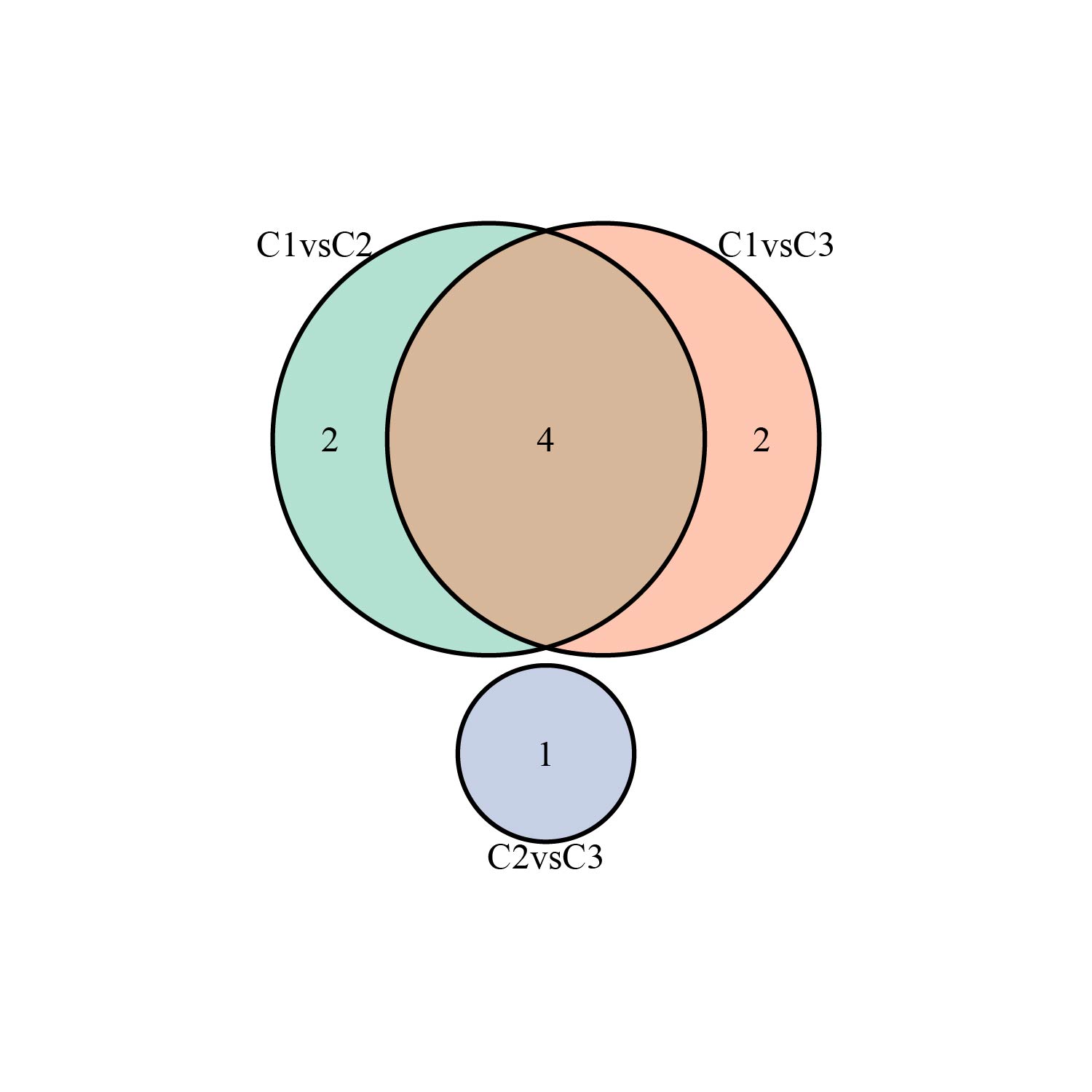

Supplement: Supplementary Figure 2 — The Venn diagram shows the the number of DEGs between each of the two molecular subtypes. [file Image_2.jpeg]

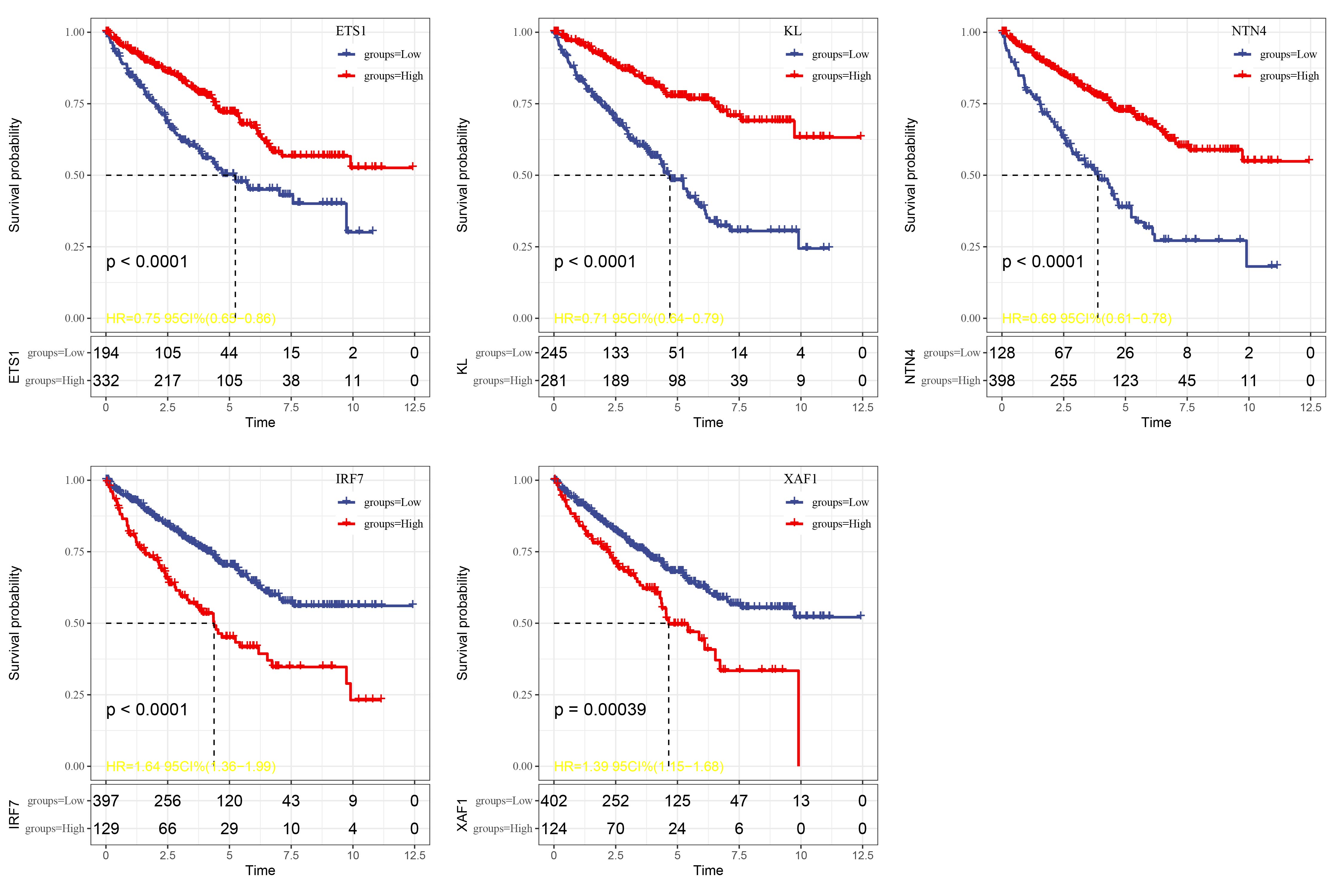

Supplement: Supplementary Figure 3 — Survival curves based on the expression of each CS-related gene in the risk model. [file Image_3.jpeg]

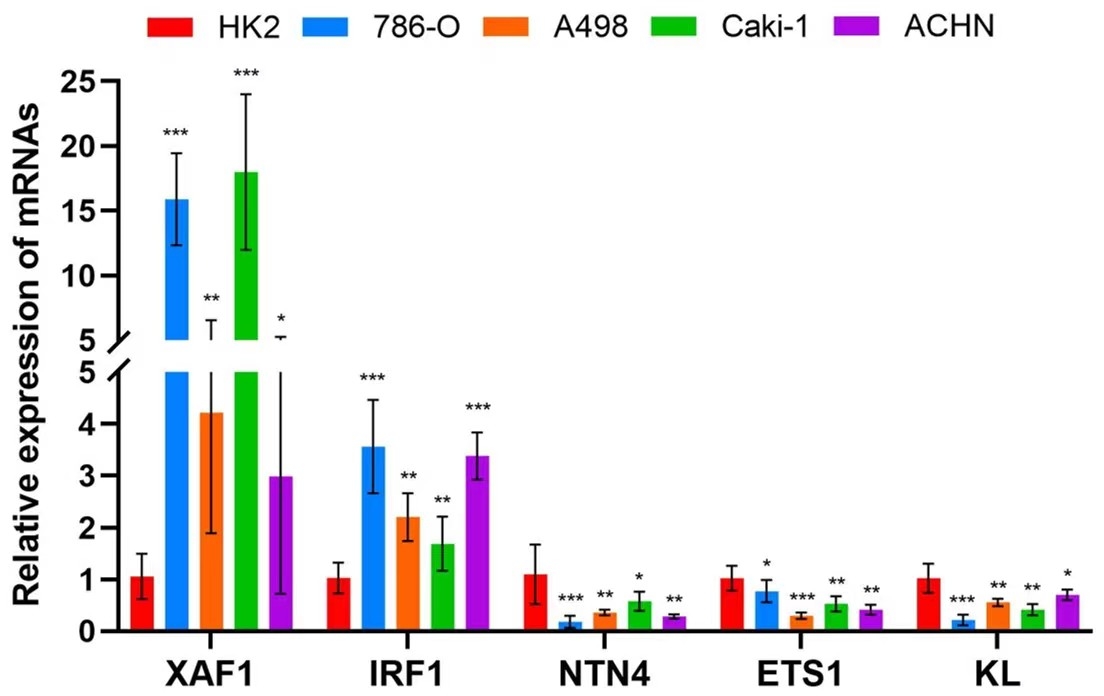

Supplement: Supplementary Figure 4 — mRNA levels of five genes in the risk model in normal kidney cells and ccRCC cells measured by qRT-PCR. Data are shown as mean ± SD, *p<0.05, **p<0.01, ***p<0.001. [file Image_4.jpeg]
